# Sema4C Is Required for Vascular and Primary Motor Neuronal Patterning in Zebrafish

**DOI:** 10.3390/cells11162527

**Published:** 2022-08-15

**Authors:** Jiajing Sheng, Boxuan Jiang, Ruijun Shi, Linsheng Shi, Dong Liu

**Affiliations:** 1 Key Laboratory of Neuroregeneration of Jiangsu and Ministry of Education, Co-Innovation Center of Neuroregeneration, Second Affiliated Hospital, School of Life Science, Nantong University, Nantong 226001, China; 2 School of Life Sciences, Wuhan University, Wuhan 430072, China

**Keywords:** endothelial cells, guidance cues, motor neuron, pathfinding, zebrafish

## Abstract

Endothelial cells (ECs) and neurons share a number of common signaling pathways and molecular mediators to orchestrate directional migration and guide the pattern of the vascular network and nervous system. So far, research concerning the functional coupling between vascular and neuronal pathfinding remains insufficient. Semaphorin4C (sema4C), a member of class 4 semaphorins, is initially described in the nervous system, whose role has been demonstrated in diverse biological developments. The present study focused on the role of *sema4C* in the vascular and neural development process in zebrafish embryos. It confirmed that *sema4C* is expressed in both the nervous system and intersegmental vessels (ISVs) in zebrafish embryos by diverse expression analysis. It also showed that the knockdown of *sema4C* caused a serious pathfinding anomaly both in the ISVs and primary motor neurons (PMNs) of zebrafish embryos. In addition, overexpressing exogenous *sema4C* mRNA in *sema4C* morphants remarkably neutralized the defective pattern of the vascular and neural system. Collectively, this report suggests that *sema4C* acts as a dual guiding factor regulating vascular and neuronal development. These findings elucidate a new molecular mechanism underlying blood vessel and nerve development and might serve as groundwork for future research on functional coupling between both systems.

## 1. Introduction

During vertebrate embryogenesis, the structural pattern of the blood vessels is essential for proper functioning [1]. Vascular development occurs under normal physiological and pathological conditions. Abnormal blood vessels are closely associated with a variety of diseases, such as atherosclerosis, stroke, diabetic retinopathy, rheumatoid arthritis and cancer [2]. Therefore, angiogenesis has been considered as an important therapeutic target for related diseases. Although research on vascular development has made significant advances, the detailed understanding of the regulation mechanisms remains greatly unexplored [3,4,5]. Vessels and nerves intersect in the process of development, and several recent studies suggested that axon-guiding signals may be involved in angiogenesis [6,7]. Therefore, research direction concerning vascular development by axon cues is expected to discover new regulation mechanisms of angiogenesis.

Semaphorins are a large family of secreted and transmembrane proteins, originally studied as axon guidance in the developing nervous system [8,9]. There are eight subfamilies of semaphorins, among which class 4 semaphorins (sema4) are one of the largest [10]. Members of sema4 have been well characterized as important regulators in diverse biological events. Sema4D/CD100 have critical roles in the immune system [11], nervous development [12,13], tumor progression [14], angiogenesis [14,15] and skeletal muscle development [16]. Sema4E is necessary for guiding branchiomotor axons to their targets in zebrafish [17]. Semaphorins4C (sema4C) is also an important member of sema4, which has been extensively studied in nerve development. sema4C mutant mice displayed obvious cerebellar defects, leading to neonatal death [18]. Furthermore, studies have demonstrated that sema4C was also implicated in biological events outside the nervous system, such as cell migration [19], tumor development [20], and terminal myogenic differentiation [21]. Furthermore, sema4C was found to promote angiogenesis in breast cancer [20]. sema4C and its preferential receptor Plexin B2 are widely expressed in discrete cell types of several nonneuronal tissues, including endothelia and endocrine cells of mouse [22,23]. The abovementioned studies indicate that sema4C may participate in endothelial cell behaviors. However, the physiological role of sema4C in vascular development remains unexplored.

Zebrafish have unique advantages in tracking endothelia cell behavior and vascular development in vivo by expressing fluorescent proteins in corresponding cells [24]. To study the roles of *sema4C*, the sequence homology, spatial expression pattern and developmental morphology of blood vessels and primary motor neurons caused by *sema4C* deletion were identified by using the unique benefits of the zebrafish model [24,25]. Current research results demonstrate that *sema4C* play dual functions in the development of endothelia cells and primary motor neurons in zebrafish. The study might serve as groundwork for future research to elucidate the extent of molecular mechanisms involved in vascular and nerve development.

## 2. Materials and Methods

### 2.1. Zebrafish Husbandry

All adult zebrafish (*Dario rerio*) were maintained at 28.5 ± 1 °C with 14 h photoperiod in the tank (1550 mm × 550 mm × 2000 mm) of a breeding system (ESEN, Beijing, CHN) [26,27]. The zebrafish were fed artemia twice a day. The wild type zebrafish (*AB/WT)* and two transgenic zebrafish lines labeling the endothelial cells (*Tg(kdrl:ras-mCherry)*) and motor neurons (*Tg(mnx1:EGFP)*), respectively, were utilized in this work [28]. The construction of the transgenic zebrafish line *Tg(fli1a:cas9-2A-EGFP)* followed the Lifetech Multisite Gateway Manual (Life Technologies, Carlsbad, CA, USA). The middle entry vector (pME-Cas9-T2A-EGFP) was obtained from Addgene (#63155).

### 2.2. Fluorescence-Activated Cell Sorting (FACS)

*Tg(fli1a:EGFP)* zebrafish embryos were cultured to 72 hpf and dechorionated by forceps. A total of 300–400 embryos were used for cell collection and then analyzed by flow cytometer [29]. The cells with EGPF fluorescence were target cells.

### 2.3. RNA Extraction and Gene Expression Analysis by Quantitative Real Time PCR (qRT-PCR)

Total RNA was isolated from zebrafish embryos (*n* = 10) at various stages (24 hpf, 48 hpf, 72 hpf and 96 hpf) and from cells separated via FACs using TRizol (Invitrogen, Waltham, MA, USA), respectively. Then cDNA was synthesized using Transcriptor First Strand cDNA Synthesis Kit (Roche) and saved at −20 °C. Quantitative RT-PCR was carried out using the following primers:

*sema4C*-QF: 5′-TGACGCCACGCTCAACTT-3′;
*sema4C*-QR: 5′-TCCGCTGTGCCTATGAAGAG-3′.

### 2.4. Whole Embryo In Situ Hybridization

Whole embryo in situ hybridization (WISH) was carried out in accordance with our published methods [27]. The sema4C fragment was cloned from cDNA using the following primers:

*sema4C*-probe-F: 5′-AATGTGACAGTGGTCGTTGG-3′;
*sema4C*-probe-R: 5′-AGCCGTCTGAGCAGTAGTAAT-3′.

Then, the fragment was inserted into pGEM-T-easy vector and digoxigenin-labeled antisense probes were synthesized. The stages of the embryo were defined as described previously [30]. Zebrafish embryos were harvested at different periods, and the chorion was removed mechanically on 48 hpf. The embryos were treated overnight in 4% PFA/PBS, washed with 1X PBST, dehydrated in successive methanol (25% MetOH/PBS, 50% MetOH/PBS and 75% MetOH/PBS, respectively), and then saved at 4 °C. The step of WISH and sectioned histological analysis followed our previous description [28,31].

### 2.5. Morpholino and mRNA Injections

Dilutions (0.3 mM) of splicing-blocking Morpholino (Gene Tools, 5 ng): 5′-CTTTTCTTGTCTGAACATAC CTGTG-3′ that were specific for sema4C gene were injected into the 1-cell embryos of *Tg(kdrl:ras-mCherry)* and *Tg(mnx1:EGFP)* zebrafish [28].

### 2.6. sgRNA/Cas9 mRNA Synthesis and Injections

Cas9 mRNA was made by in vitro transcription following our published steps [32]. The *sema4C* guide RNA (gRNA) (5′-AGCCGATCACGAAGATCACAAGG-3′) was prepared and made according to our previously described procedure [33]. In addition, *sema4C*-gRNA was injected into the embryos of *Tg(fli1a:cas9-2A-EGFP)* to construct tissue-specific knockout zebrafish. Approximately 2–3 nL liquor, including Cas9 mRNA and sgRNA was injected into 1-cell embryos [34]. Then, these embryos were collected for genomic DNA extraction at 72 hpf [34]. Next, the measure efficiency of cutting was measured by sequencing using primers as follows:

*sema4C*-cas9-F: 5′- GTGCGAAAGTTCTCCTTGTGA -3′;
*sema4C*-cas9-R: 5′-AACATGGTGTAGTTCCACACC -3′.

### 2.7. mRNA Rescue Experiments

The full-length of *sema4C* (ENSDART00000113037.3) was cloned into PCS2+ vector and transcribed in vitro with the mMESSAGE mMACHINE Sp6 Ultra Kit (Ambion, Austin, TX, USA). The Approximately 2 nL of the mixture containing purified mRNA, and sema4C Mo was then injected into 1-cell embryos.

### 2.8. Microscopic and Statistical Analysis

The anesthetized embryos were placed in low-melt agarose followed by imaging under a confocal microscope (AID2, NIKON, Tokyo, Japan). The pictures of WISH were taken with the stereomicroscope (MVX10, Olympus, Tokyo, Japan). One-way analysis of variance (ANOVA) and the Mann–Whitney test were used for statistical analysis. If *p*-value < 0.05, the statistical difference is considered significant.

## 3. Results

### 3.1. sema4C Genes Are Highly Conserved in Vertebrates

**Figure 1:**
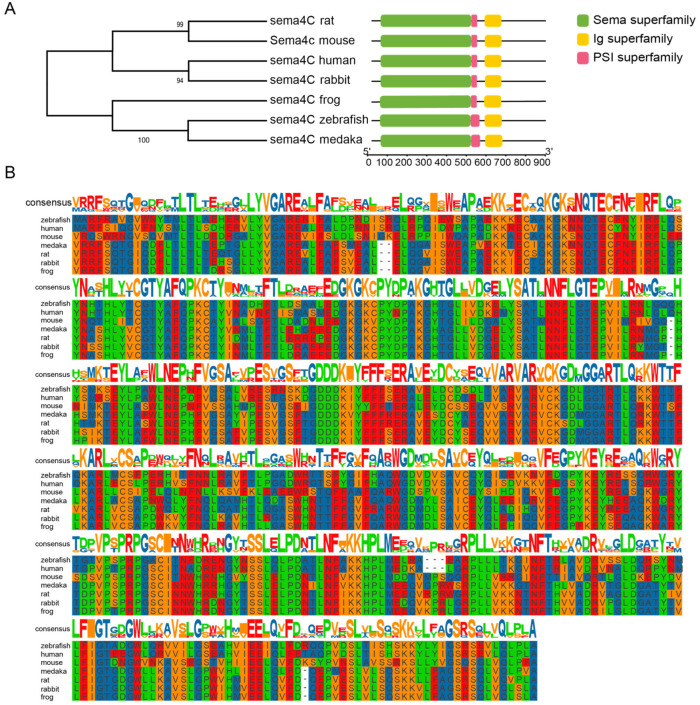
**sema4C is highly conserved during evolution.** (**A**). Phylogenetic tree and domain structure of sema4C protein; (**B**). Alignment of the protein sequences of the sema domain of sema4C in Danio rerio, Homo sapiens, Mus musculus, Oryzias latipes, Rattus norvegicus, Oryctolagus cuniculus and Xenopus tropicalis. These protein sequences were aligned using MEGA7 software and edited by TBtools.

We examined sema4C protein phylogeny in zebrafish (NP_001121718.1) and other representative species, including medaka (*Oryzias latipes*, NC_019867.2), frog (*Xenopus tropicalis*, NC_030679), rabbit (*Oryctolagus cuniculus*, NC_013670.1), mouse (*Mus musculus*, NC_000067), rat (*Rattus norvegicus*, NC_051344.1) and human (*Homo sapiens*, NC_000002.12). The results of the phylogenetic tree show that the *sema4C* from zebrafish and medaka were clustered in different clades from rodents and primates (Figure 1A). In addition, the *sema4C* proteins are highly conserved during evolution, especially the sema domain, which suggests their important functions (Figure 1B).

### 3.2. Expression of sema4C Genes in Zebrafish

**Figure 2:**
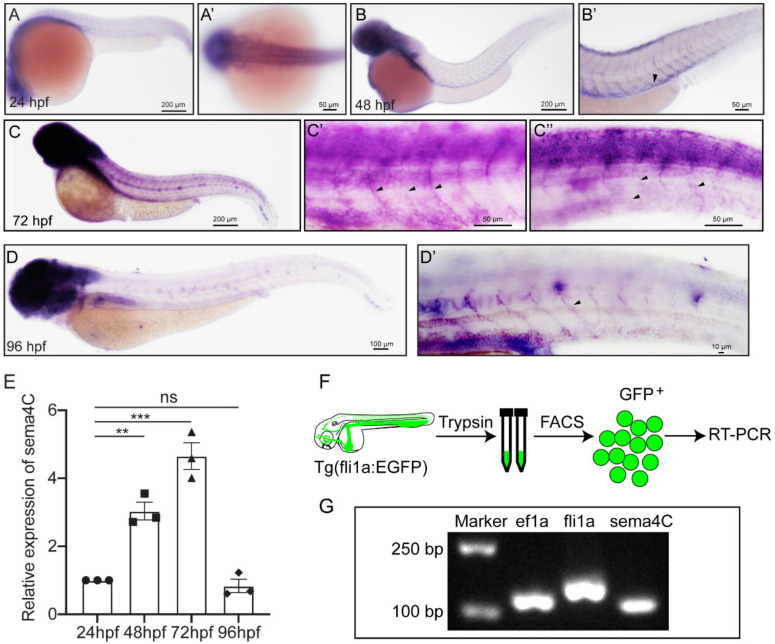
**Expression of *sema4C* gene in embryonic zebrafish at different stages.** (**A**). Whole mount in situ hybridization (WISH) analysis of *sema4C* in zebrafish embryos and the magnified image of the head at 24 hpf (**A’**); (**B**–**D**). WISH analysis of *sema4C* in zebrafish embryos and the magnified images of the trunk at 48 hpf (**B’**), 72 hpf (**C’**,**C”**) and 96 hpf (**D**,**D’**), black arrow-heads indicate blood vessels; (**E**). Expression analysis of *sema4C* genes in embryonic zebrafish at different stages by QRT-PCR. Triangles, squares, dots and diamonds represent data at different time points respectively. One-way ANOVA, *** *p* < 0.001, ** *p* < 0.01, ns, no significance; (**F**). The procedure of the endothelial cells sorting and RT-PCR; (**G**). The agarose gel electrophoresis results of RT-PCR on *fli1a-EGFP* sorted cells.

We investigated the expression levels of *sema4C* using QRT-PCR and WISH. The results of QRT-PCR show that sema4C was continuously expressed from 24 hpf to 96 hpf, with the highest expression at 72 hpf (Figure 2E). We further studied the expression patterns of *sema4C*, and found that *sema4C* was mainly expressed in the brain and head structures (Figure 2A–D). Additionally, *sema4C* was also observed to be expressed in intersegmental vessels (ISVs) in 48 (Figure 2B’) and 72 hpf embryos (Figure 2C’,C”). In order to further examine the expression of *sema4C* in zebrafish blood vessels, we sorted endothelial cells from *Tg(fli1a:EGFP)* and then performed RT-PCR (Figure 2F). The results show that both *fli1a* and *sema4C* could be detected in the sorted endothelial cells (Figure 2G). Together, these results suggest that *sema4C* may play dual roles in zebrafish vascular and nervous system development.

### 3.3. sema4C Deficiency Caused Aberrant Axonal Projection of PMNs

**Figure 3:**
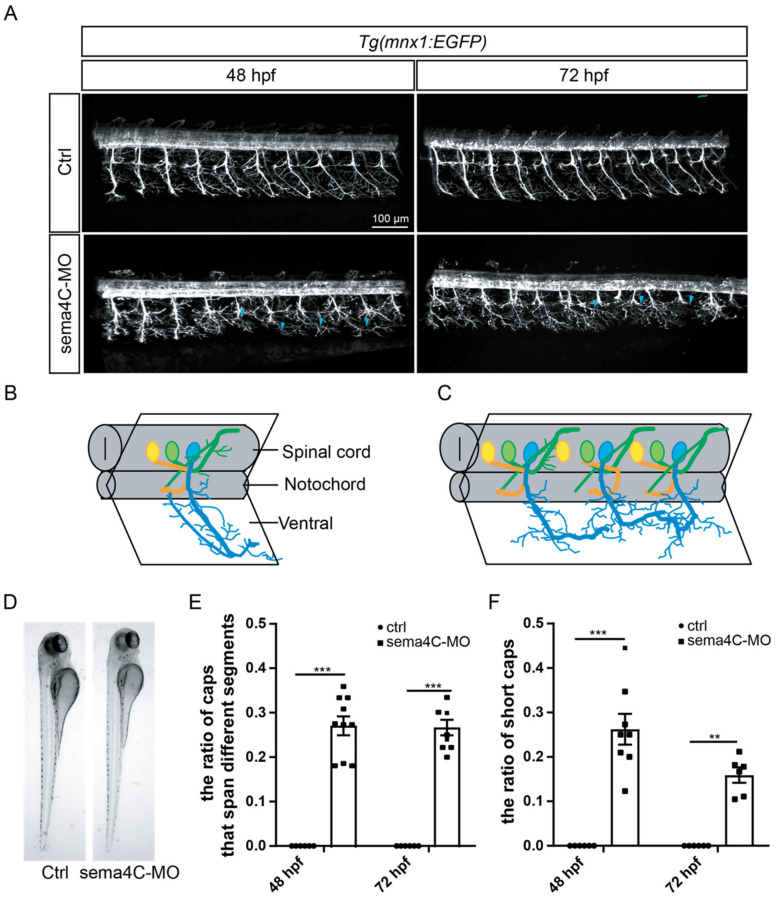
**The abnormal development of primary motor neurons in the *sema4C* deficiency zebrafish.** (**A**). Confocal imaging analysis of primary motor neurons in control and *sema4C* knockdown *Tg(mnx1:EGFP)* zebrafish at 48 and 72 hpf, blue arrowheads indicate aberrant Caps; (**B**). The schematic diagram for three different primary motor neurons in the control fish; (**C**). The schematic diagram for the abnormal pattern in the sema4C morphants, in which the axonal trajectories of Caps were dramatically misled; (**D**). Zebrafish embryos of control and *sema4C*-MO injected at 72 hpf imaged in bright field; (**E**). The statistical analysis of the ratio of Caps across different segments in the control and *sema4C* morphants at 48 and 72 hpf. Mann–Whitney test, 48 hpf: *** *p* = 0.0007; *** 72 hpf: *p* = 0.0007; (**F**). The statistical analysis of the ratio of short Caps in the wild types and *sema4C* morphants at 48 and 72 hpf. Mann–Whitney test, 48 hpf: *** *p* = 0.0007; 72 hpf: ** *p* = 0.0022.

sema4C is expressed throughout the nervous system during the development of zebrafish, and particularly highly expressed in the basal parts of the fore-, mid- and hindbrain [35]. Therefore, it is reasonable to speculate it may regulate the axonal projection of neurons. To determine the roles of *sema4C* during zebrafish motor neuron development, specific morpholino antisense oligonucleotide (sema4C-MO) was used to knock down the expression of *sema4C*. The results of RT-PCR proved that the *sema4C*-MO could efficiently disrupt the exact transcription of *sema4C* (Supplementary Figure S1). Confocal microscopy analysis demonstrated that the deficiency of *sema4C* resulted in significant developmental defects of PMNs at 48 and 72 hpf (Figure 3A), while these injected zebrafish embryos were generally normal (Figure 3D). In particular, the axonal trajectories of caudal primary motor neurons (Caps) were dramatically misled in the *sema4C* morphants (Figure 3C,E), while the wild types of axons projected to the horizontal myoseptum in an ordered pattern (Figure 3B). In addition, partial PMNs in *sema4C* morphants were significantly shorter in length and could not be completely recovered until 72 hpf (Figure 3F). Overall, these findings show that *sema4C* is necessary for the development and navigation of neural networks.

### 3.4. sema4C Deficiency Leads to Abnormal Vascular Pathfinding in Zebrafish

**Figure 4:**
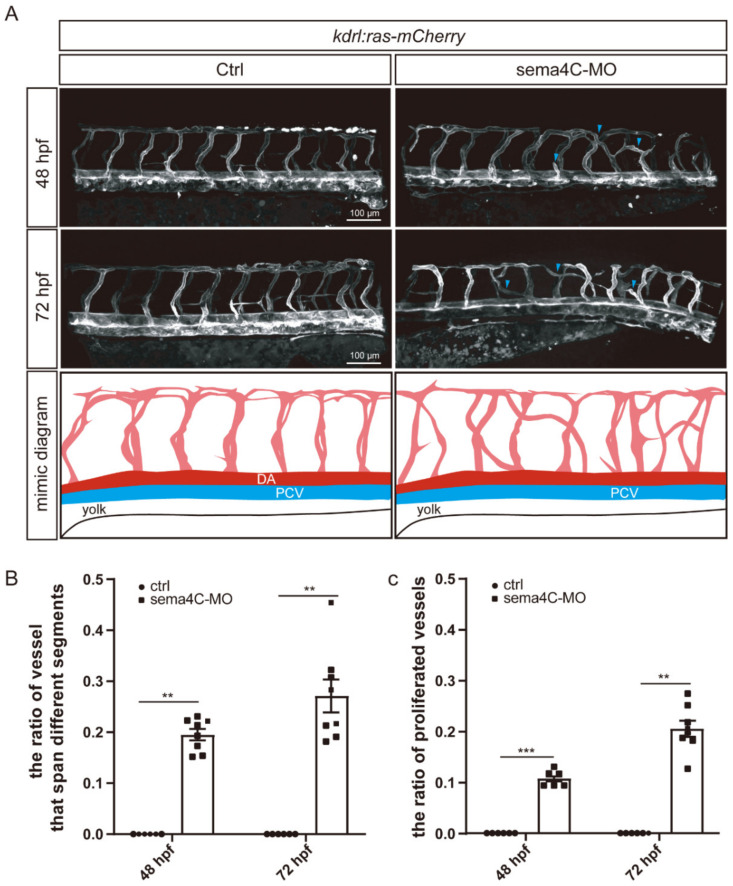
**Deficiency of *sema4C* leads to abnormal vascular networks.** (**A**). Confocal imaging and the schematic diagram of ISVs in the control and *sema4C*-MO *Tg(kdrl:ras-mCherry)* zebrafish at different stages. As shown in the diagram, ISVs in the control group were orderly arranged and grew upwards, while ISVs’ growth turned to horizontal germination and connected to adjacent ISVs, or even the opposite ISVs. In sema4C knockdown of zebrafish, blue arrowheads indicate aberrant ISVs; (**B**). The statistical analysis of the ratio of vessels that crossed different segments in the control and *sema4C* morphants at different stages—Mann–Whitney test, 48 hpf: ** *p* = 0.0012; 72 hpf: ** *p* = 0.0012; (**C**). The statistical analysis of proliferated vessel ratio in the control and *sema4C* morphants at different stages—Mann–Whitney test, 48 hpf: *** *p* = 0.0006; 72 hpf: ** *p* = 0.0012.

Because we found that *sema4C* was expressed in zebrafish vessels, it is reasonable to speculate that it may modulate the formation of the vascular system. In order to further investigate the role of *sema4C* during the development of vessels, the morphogenesis of ISVs was also examined in the *sema4C* knocking down zebrafish at different stages (Figure 4A). Consistent with motor neuron development, the deficiency of *sema4C* led to significant pathfinding defects of zebrafish ISVs. The ISVs in *sema4C* knockdown zebrafish grew upwards to the middle segment, and then turned to horizontal sprouting and connected to adjacent ISVs, even opposite ISVs (Figure 4B). In addition, the generation of proliferated vessels was found in the *sema4C*-deficient zebrafish (Figure 4C). These findings suggest that *sema4C* also appears to be essential for vascular pathfinding.

### 3.5. The Aberrant Patterns of Both Nerves and Vascular System Were Confirmed in sema4C F0 Knockouts

**Figure 5:**
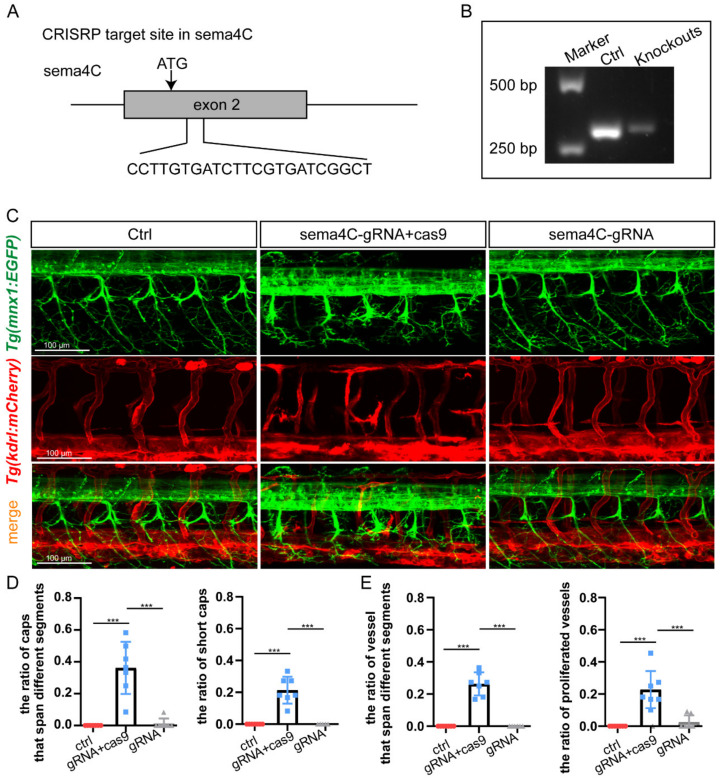
**Knockout of *sema4C* caused both aberrant phenotypes in PMNs and ISVs.** (**A**). The targeting site of the gRNA on the *sema4C* gene; (**B**). The measure of cutting efficiency using genomic DNAs of injected embryonic (*n* = 10); (**C**). Confocal imaging analysis of PMNs and ISVs in control and the F0 generation of the injected *Tg(mnx1:EGFP::kdrl:ras-mCherry)* zebrafish at 48 hpf, blue arrowheads indicate aberrant PMN and ISV; (**D**). The statistical analysis of the proportion of abnormal axonal trajectories of Caps (*** *p* = 0.0006) and short Caps (*** *p* = 0.0006) in the different groups at 48 hpf. Mann–Whitney test; (**E**). The statistical analysis of the proportion of vessels that extend across different segments (*** *p* = 0.0006) and the proportion of proliferated vessels (*** *p* = 0.006) in the different groups at 48 hpf. Mann–Whitney test.

To further prove that *sema4C* is necessary for PMNs and ISVs pathfinding, the CRISPR/Cas9 system was used to knockout *sema4C* in *Tg(mnx1:EGFP::kdrl:ras-mCherry)* zebrafish. The target sites near or downstream of the translation start codon (ATG) of *sema4C* coding sequence were selected for gRNAs design to ensure the functional proteins were completely destroyed (Figure 5A). The result of CRISPR editing efficiency measurement using injected embryonic genomic DNAs (*n* = 10) indicated a high efficiency of the selected Cas9 system (Figure 5B). Confocal imaging analysis showed that the morphogenesis of PMNs and ISVs in the F0 *sema4C* knockouts was in accordance with that in morphants (Figure 5C). Specifically, the development of PMNs was impaired and the axonal trajectories were clearly disordered in the F0 knockouts (Figure 5D). Further, a disorganized vascular system was also found (Figure 5E). In addition, the injection of *sema4C* gRNA without cas9 did not lead to significant developmental defects, proving that the phenotype was specifically caused by *sema4C* knockout.

### 3.6. Overexpressing sema4C Partially Rescued the Abnormal Phenotypes of ISVs and PMNs in sema4C Deficient Embryos

**Figure 6:**
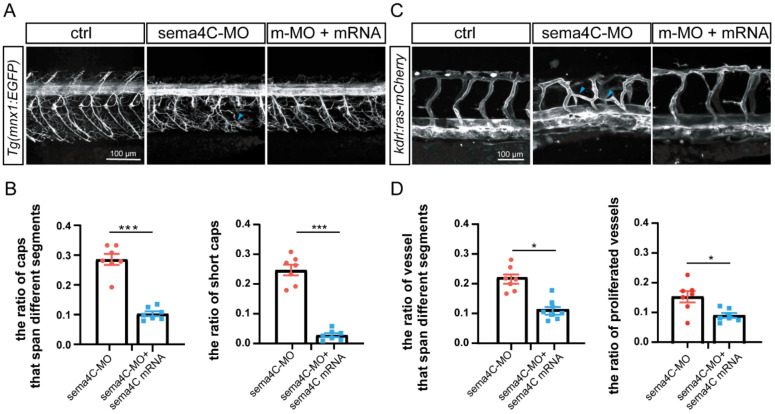
**Overexpressing *sema4C* partially rescued the abnormal of PMNs and ISVs in *sema4C* deficient embryos.** (**A**). Confocal imaging analysis of PMNs in control and sema4C morphants at 72 hpf, blue arrowheads indicate aberrant PMNs and ISVs; (**B**). The statistical analysis of the proportion of deficient axonal trajectories of Caps (*** *p* = 0.0006) and short Caps (*** *p* = 0.0006) in the *sema4C* morphants and *sema4C* rescue groups at 72 hpf. Dots and squares represent the data of the experimental group and the rescue group, respectively. Mann–Whitney test; (**C**). Confocal imaging analysis of ISVs in control and *sema4C* morphants at 48 hpf; (**D**). The statistical analysis of the ratio of vessels that span different segments (* *p* = 0.035) and the proportion of proliferated vessels (* *p* = 0.0262) in the *sema4C* morphants and sema4C rescue groups at 48 hpf. Dots and squares represent the data of the experimental group and the rescue group, respectively. Mann–Whitney test.

To further validate that the vascular and neuronal defects were caused by the lack of specificity of *sema4C*, *sema4C* mRNA and *sema4C*-Mo were co-injected into zebrafish embryos. Then, we randomly selected 7–8 embryos to examine ISVs and PMNs morphogenesis by confocal imaging analysis. The results are strongly in line with our expectations: the *sema4C* mRNA co-injection greatly normalized the defective phenotypes of PMNs (Figure 6A,B) and ISV projections (Figure 6C,D) in *sema4C*-deficient embryos. In addition, the growth restriction of PMNs observed in sema4C morphants was also rescued. These results confirm that sema4C could regulate both vascular and primary motor neuronal patterning in parallels in zebrafish.

## 4. Discussion

Semaphorins, a large family of proteins, are expressed in most organs and tissues. Previous studies reported that semaphorins members play important regulatory roles in the development of the nervous system (Sema3A, 3F, 4D, 6C, 6D, 7A), immune system (Sema4D), reproductive systems (Sema3), cancer progression (Sema3A, 4D, 6D) and the vascular system (Sema3A, 3E, 4D, 6D) [36,37,38,39,40]. sema4C belongs to class 4 semaphorins, which were initially reported to participate in the signal pathway involved in neuronal migration. However, our understanding of the roles of *sema4C* in motor neuron development or blood vessel formation is still limited. Taking advantage of the zebrafish model to explore the role of *sema4C* in embryogenesis is significant for understanding neuro-vascular communication. The present data on *sema4C* phenotype of *sema4C*-deficient zebrafish provide new insights into the roles of semaphorin families in the nervous and the vascular system.

Firstly, it was confirmed that *sema4C* is expressed in the nervous system as well as ISVs of zebrafish embryos by in situ hybridization. Next, the Morpholino knockdown of *sema4C* disrupted the construction of ISVs and PMNs, suggesting that *sema4C* might guide endothelial cell and neuron navigation during zebrafish embryogenesis. Furthermore, the overexpression of *sema4C* partially restored the development of vessels and neurons in *sema4C* morphants, confirming that the deficiency in phenotypes was the result of down-regulation of *sema4C*. Current data demonstrates that *sema4C* plays an important role in the development and construction of neural and vascular systems in zebrafish embryos.

It has been reported that *sema4C* is expressed in the adult brain [41]. Soluble sema4C could increase the adhesion of the granule cell precursors to laminin and stimulate their proliferation and migration. *sema4C* mutant mice displayed significant defects of the cerebellar granule cell layer [19]. In addition, *sema4C* is generically expressed in the zebrafish embryo, including the central nervous system, cardiovascular system, olfactory neural system, optic nerve system, notochord, pectoral fin and pharyngeal arch [22]. In this study, our results of in situ hybridization also confirm that *sema4C* is enriched in the embryonic brain of zebrafish. It is not surprising that zebrafish acquired abnormal neuronal phenotypes due to the deficiency of *sema4C*, which was consistent with previous studies in other species [19,42]. However, unexpected findings were the aberrant patterning of the primary motor neurons, which has not been reported in previous studies. Our findings suggest that *sema4C* is enriched in the zebrafish nervous system and responsible for the developmental defects of PMNs.

Different guidance clues for the dynamic regulation of endothelial cells behavior coordinate the precise wiring of the complicated vascular system [6,7,8]. At present, more and more evidence has shown that the organization of the blood vascular system share similar or common cues with neural networks [36,43]. Except in the nervous system, studies demonstrated that sema4c was implicated in other biological processes, including cell migration, tumor development, terminal myogenic differentiation and EMT [20,44]. In our previous study, *sema4C* was found to be significantly expressed in endothelial cells of zebrafish by single-cell sequencing [45]. Here, we found that *sema4C* was expressed in the ISVs of zebrafish. Next, the knockdown and knockout of *sema4C* in the zebrafish embryo was found to cause dramatic pathfinding abnormal in ISVs. Furthermore, the specific knockdown of *sema4C* in ECs also led to the impairment of vascular pathfinding, but did not affect neurons. These data suggest that *sema4C* can modulate vascular patterns in zebrafish development.

To date, more and more studies indicate that endothelial cells and neurons share a variety of common guiding clues in participate in the patterns of complex vascular and neuronal systems. Nevertheless, knowledge regarding the functional overlap between vascular and neuronal systems is still limited, calling for more advanced research. The present study identified that *sema4C* is expressed in both ISVs and the nervous system. *sema4C*, as a guidance signal, is involved in regulating the branching and pathfinding of blood vessels and neurons during zebrafish development. Although more details of the functional mechanism of the *sema4C* genes still need to be determined in future studies, these findings are nevertheless helpful for elucidating a new molecular mechanism underlying the nerves and vascular development.

## Data Availability

All the experimental materials generated in this study are availble from the corresponding authors upon reasonable request.

## Supplementary Materials

The following supporting information can be downloaded at: https://www.mdpi.com/article/10.3390/cells11162527/s1, Figure S1: sema4C-MO effectively reduced the expression of sema4C through splice blocking. A. RT-PCR analysis of sema4C on AB embryos and sema4C-MO injected embryos; B. The schematic diagram of sema4C transcripts in wild embryos; C. The schematic diagram of sema4C transcripts in sema4C-MO injected embryos.
