# Supplementary material for: Sema4C Is Required for Vascular and Primary Motor Neuronal Patterning in Zebrafish
Source: Cells. 2022 Aug 15;11(16):2527. doi: 10.3390/cells11162527 (PMC9406964; doi:10.3390/cells11162527)
Supplement: Supplementary file 1 [file cells-11-02527-s001.zip › cells-1810463-supplementary.pdf]

Supplementary data

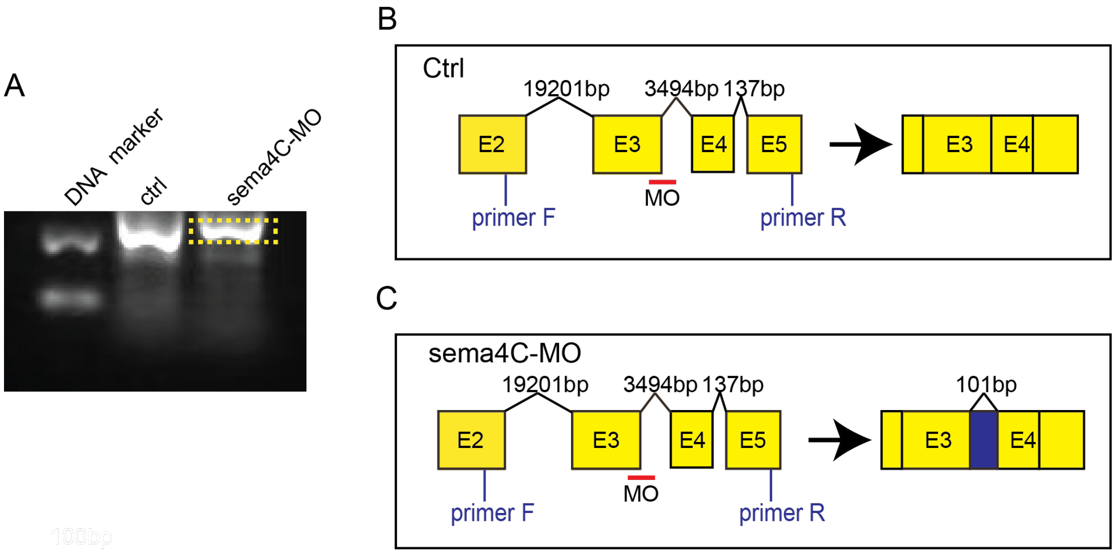

Figure S1. sema4C-MO effectively reduced the expression of sema4C through splice blocking. A. RT-PCR analysis of sema4C on AB embryos and sema4C-MO injected embryos; B. The schematic diagram of sema4C transcripts in wild embryos; C. The schematic diagram of sema4C transcripts in sema4C-MO injected embryos.
